# Atypical Cutaneous Manifestation of Mycosis Fungoides: A Case Report

**DOI:** 10.7759/cureus.65034

**Published:** 2024-07-21

**Authors:** Veronica Hagan, Elisha Myers, Thomas Caussat, Abid Sobhan, Luisa Barrueto, Areeba Awan

**Affiliations:** 1 Medicine, Florida Atlantic University Charles E. Schmidt College of Medicine, Boca Raton, USA; 2 Internal Medicine, Florida Atlantic University Charles E. Schmidt College of Medicine, Boca Raton, USA; 3 Internal Medicine, Boca Raton Regional Hospital, Boca Raton, USA

**Keywords:** mycosis fungoides, skin microbiota, atypical manifestation, bacterial superinfection, primary cutaneous t-cell lymphoma

## Abstract

Mycosis fungoides (MF) is a cutaneous T-cell lymphoma (CTCL) that is characterized by atypical CD4+ T-cell aggregates in the epidermis. It is typically divided into three clinical phases, which consist of the patches, plaques, and tumor stages. There have been atypical manifestations of MF described in the literature, and it is hypothesized that the skin microbiota plays a role in the skin phenotype of MF patients. Here, we describe an MF patient with multiple, large, ulcerated, and purulent lesions that developed after she swam in the ocean. Our patient was found to have a unique set of bacteria isolated from the wound.

## Introduction

Mycosis fungoides (MF) is the most common type of cutaneous T-cell lymphoma, occurring in approximately 1 in 100,000 to 350,000 individuals, predominantly affecting middle-aged and elderly males [1]. MF progresses through three clinical phases: the patches, plaques, and tumor stages. Lesions commonly appear on the lower abdomen, upper thighs, buttocks, and breasts. These lesions can later develop mushroom-shaped tumors within the plaques, contributing to the disease's name [2]. The diagnosis of MF involves a biopsy of the lesions, revealing atypical CD4+ T-cells with cerebriform nuclei in the dermis and epidermis, along with Pautrier abscesses [2]. Treatment options vary depending on the stage, with local corticosteroids and psoralen combined with ultraviolet A irradiation being common for initial treatment. Advanced stages may require chemotherapy or allogeneic stem cell transplant [3]. Here, we present a unique case of MF featuring atypical lesions and a superimposed infection of *Morganelli morganii*, *Pseudomonas aeruginosa*, *Enterococcus avium,* and *Enterococcus faecalis*.

## Case presentation

**Figure 1:**
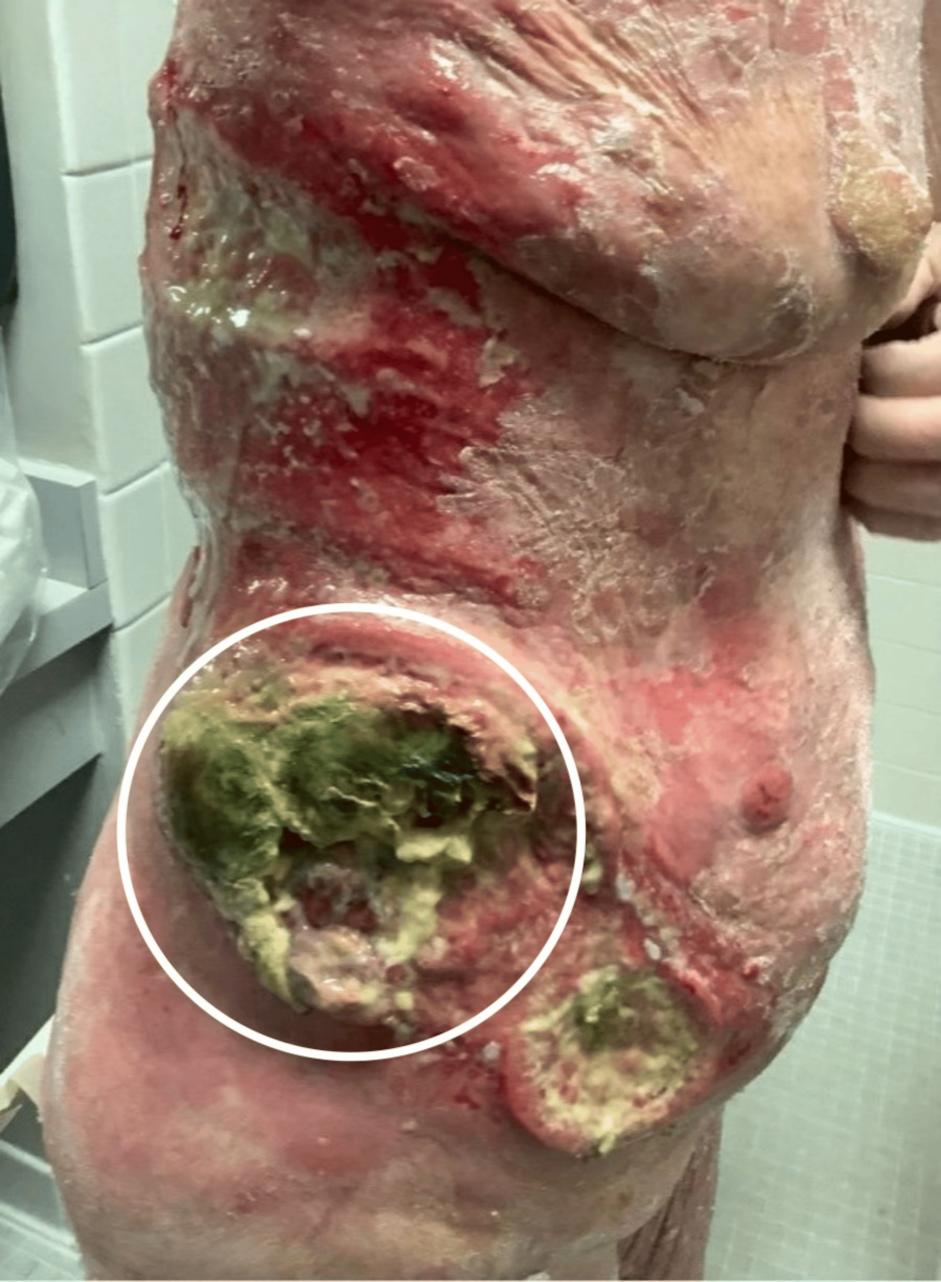
Two large erythematous round lesions that were actively purulent on her right flank

**Figure 2:**
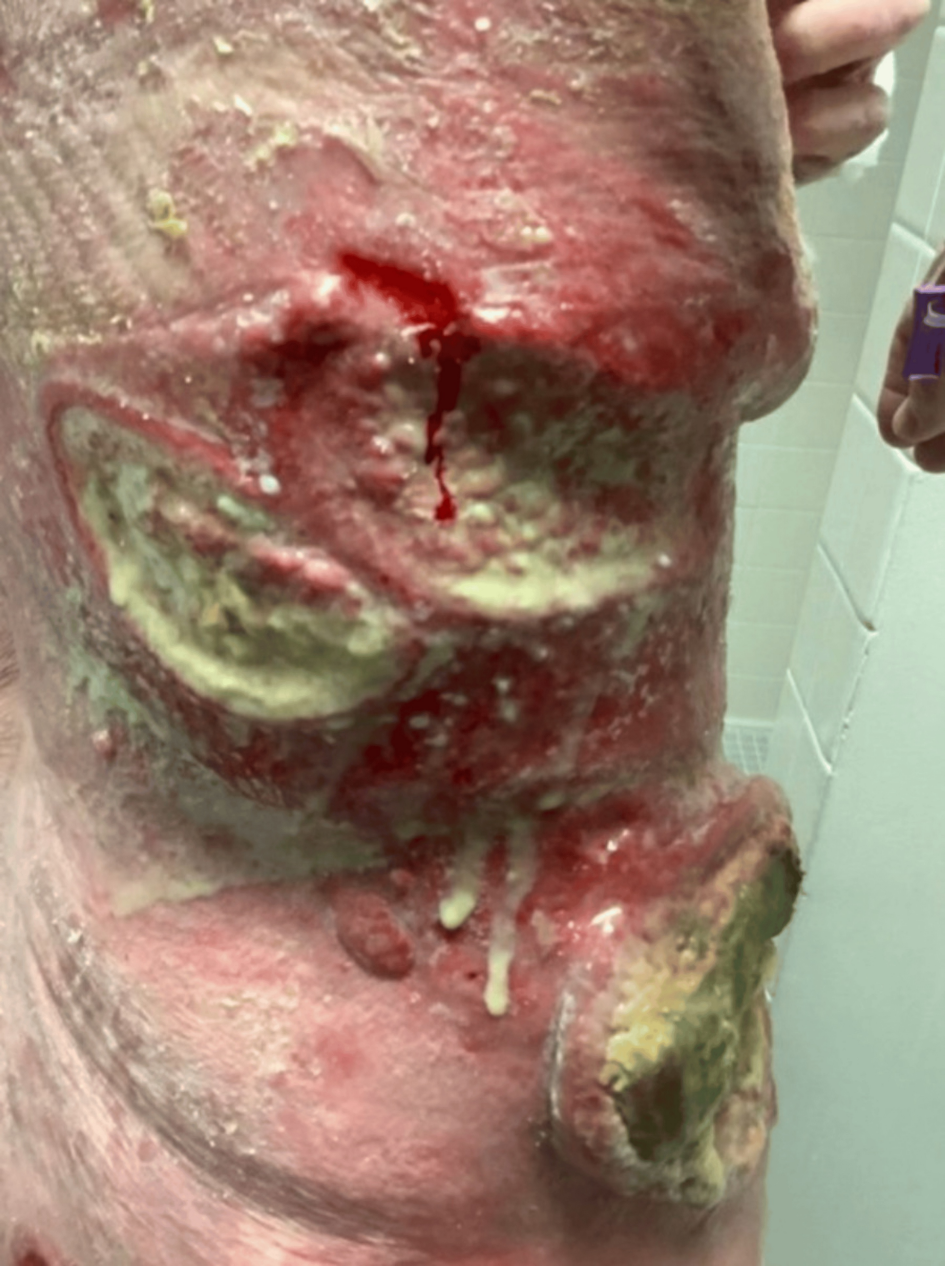
Purulent lesions overlying plaques on the patient’s back and right flank

A 68-year-old female with a history of MF presented to the emergency department with weakness lasting one week and painful soft tissue lesions that had been worsening over the past three months. She lived independently and was forced to call emergency medical services after falling. While her cardiovascular and pulmonary exams were unremarkable, integumentary physical examination revealed erythematous, atrophic, and hyperkeratotic lesions on her abdomen, back, proximal arms, chest, and proximal legs. Notably, she had two large erythematous circular lesions with active purulence on her right lower abdomen (Figure 1) and purulent lesions overlying plaques on her back (Figure 2).

The patient was diagnosed with MF four years ago, which at that time consisted of patches without any purulence. Her lesions were initially controlled with topical steroids, with subsequent recommendations by oncology for more targeted therapy with gemcitabine followed by bexarotene. However, she was lost to follow-up during the COVID-19 pandemic and did not receive any medications beyond topical steroids. Currently, she was not receiving any MF treatment, and her lesions had worsened over the past three months, notably after swimming in the Atlantic Ocean.

Laboratory results showed a white blood count of 13,400/mcL with an absolute lymphocyte count of 0.9x10^3/mcL, absolute neutrophil count of 10.7x10^3/mcL, and absolute eosinophil count of 0.89x10^3/mcL. Cultures of the largest lesion on the right abdomen (Figure 1, circled) grew *Morganelli morganii*, *Pseudomonas aeruginosa*, *Enterococcus avium,* and *Enterococcus faecalis*. Although the patient was also found to have blood and urinalysis cultures positive for methicillin-resistant *Staphylococcus aureus* (MRSA), MRSA was absent from all wound cultures.

Treatment involved intravenous (IV) vancomycin 1,000 mg every eight hours and IV piperacillin-tazobactam 3,375 mg every eight hours for 15 days, resulting in mild improvement in the dermatological manifestation of the disease. Surgical consultation was considered for possible debridement, but she was not deemed a surgical candidate, as her wounds were not necrotic. She was discharged on day 15 of hospitalization with instructions to initiate a gemcitabine cycle followed by bexarotene as per oncology.

## Discussion

MF is the most common cutaneous T-cell lymphoma, typically presenting with erythematous patches, plaques, and eventually tumors [1]. Diagnosis relies on a combination of clinical and histologic findings [2]. Various atypical presentations of MF have been described, including cases mimicking pyoderma gangrenosum [4].

The microbiome of the skin plays a major role in diseases of the skin and the prognosis [5]. In MF, the skin microbiota, particularly *Staphylococcus (S.) aureus* colonization, is theorized to influence skin phenotypes [6,7]. However, limited studies have explored changes in the skin microbiota of MF patients. Our case highlights a unique combination of bacteria isolated from the patient’s abdominal lesion, with *Morganelli morganii*, *Pseudomonas aeruginosa*, *Enterococcus aviumm*, and *Enterococcus* *faecalis* isolated from one lesion, a combination not previously described [8]. Additionally, our patient had MRSA bacteremia, which could have been due to an MRSA infection of one of the lesions, as not every lesion was cultured. The patient's exposure to Atlantic Ocean water likely contributed to the infection of her lesions, as ocean water can alter the skin microbiota and introduce bacteria that may cause infection, particularly in MF patients who are not taking prophylactic measures [9]. It is therefore recommended that patients cover their skin with a protective oil layer before exposure to water, with coconut oil being a commonly recommended option due to its antibacterial and anti-inflammatory properties [10,11]; however, it is not known if this would fully prevent certain bacterial infections.

Infections are common in MF patients and are one of the leading causes of death, emphasizing the importance of early detection and treatment [12]. In particular, cutaneous infections are a common cause of bacteremia in MF patients. Factors that increase one’s risk of bacteremia from a cutaneous infection include patients whose wound cultures are polymicrobial, *S. aureus*, and gram-negative infections [13]. In patients with MF who present with wound infections, wound cultures are particularly important for considering the risk of a possible superimposed bacterial infection. In one study, *Staphylococcus aureus* and *Pseudomonas aeruginosa *were found to be the most commonly isolated infectious organisms in wound cultures of patients with T-cell lymphomas [14]. Additionally, *Enterococcus* has been associated with dark eschars in MF, which respond well to antibiotics [15]. The treatment of wound infections in MF should be informed by the bacteria involved, as there can be a diverse set of bacteria isolated as seen in this case, to increase the likelihood of treatment success.

This case underscores the importance of maintaining a follow-up regimen with specialists, vigilant monitoring of lesions, and avoiding environments that may predispose patients with MF to infection. It also highlights the challenge of managing MF patients with concurrent bacterial infections involving unusual pathogens.

## Conclusions

In conclusion, this case report sheds light on an unusual presentation of MF in a 68-year-old female, which was complicated by an overlying cutaneous infection with *Morganelli morganii*, *Pseudomonas aeruginosa*, *Enterococcus avium*, and *Enterococcus faecalis*. The findings highlight the importance of maintaining a consistent follow-up regimen with specialists, closely monitoring lesions for any changes, and being cautious of environments that may predispose MF patients to infections. Furthermore, it emphasizes the complexities involved in managing MF patients, particularly when they present with concurrent bacterial infections involving uncommon pathogens, a challenge that was exacerbated during the COVID-19 pandemic. This case serves as a reminder of the critical importance of implementing proper treatment strategies and adopting prophylactic measures to mitigate the risk of cutaneous infections in MF patients.
